# Precision Laboratory Diagnostics for Autoimmune Encephalitis and Paraneoplastic Neurological Syndromes: Autoantibody Testing, Fluid Biomarkers, and AI-Assisted Integration

**DOI:** 10.3390/diagnostics16213658

**Published:** 2026-11-05

**Authors:** David Stejskal, Zdeněk Švagera, Pavlína Kušnierová, David Zeman, Petra Masarovičová, Markéta Ryzí, Jana Slonková

**Affiliations:** 1 Institute of Laboratory Medicine, University Hospital Ostrava, 708 52 Ostrava, Czech Republic; zdenek.svagera@fno.cz (Z.Š.); pavlina.kusnierova@fno.cz (P.K.); david.zeman@fno.cz (D.Z.); petra.masarovicova@fno.cz (P.M.); marketa.ryzi@fno.cz (M.R.); 2 Institute of Laboratory Medicine, Faculty of Medicine, University of Ostrava, 703 00 Ostrava, Czech Republic; 3 Lab4Life, University Hospital Ostrava, 708 52 Ostrava, Czech Republic; 4 Department of Neurology, University Hospital Ostrava, 708 52 Ostrava, Czech Republic; jana.slonkova@fno.cz; 5 Department of Neurosciences, Faculty of Medicine, University of Ostrava, 703 00 Ostrava, Czech Republic

**Keywords:** autoimmune encephalitis, paraneoplastic neurological syndromes, neural autoantibodies, neurofilament light chain, cerebrospinal fluid biomarkers, immune checkpoint inhibitors, PhIP-Seq, artificial intelligence, laboratory medicine, diagnostic accuracy

## Abstract

Autoimmune encephalitis (AE) and paraneoplastic neurological syndromes (PNS) are leading causes of potentially reversible but frequently misdiagnosed neurological disability, and delayed recognition worsens outcomes and impedes timely tumor detection. This narrative review is written from a laboratory medicine perspective and focuses on the analytical determinants of early and accurate diagnosis: appropriate test selection, dual-method confirmation combining tissue-based and cell-based assays, phenotype-guided interpretation, and assay harmonization. We summarize the expanding spectrum of neuronal and glial autoantibodies, quantitative biomarkers of neuroaxonal injury—most notably neurofilament light chain—and investigational cerebrospinal fluid markers, including cytokine profiles, kappa free light chains, and markers of astrocytic injury, whose ability to discriminate AE from infectious mimics has not been established. We highlight novel autoantibody discovery through phage immunoprecipitation sequencing, research-stage multi-omics approaches, and early, retrospectively evaluated artificial intelligence-assisted classifiers, and we address the growing challenge of neurological adverse events triggered by immune checkpoint inhibitors. Priorities for translation into routine care include external quality assurance, certified reference materials, age-adjusted reference intervals, and prospective validation of integrated biomarker panels within reference-center networks. The review provides a structured framework to support precision laboratory diagnostics in neuroimmunology.

## 1. Introduction

Autoimmune encephalitis (AE) and paraneoplastic neurological syndromes (PNS) have emerged as leading causes of subacute encephalopathy, seizures, and rapidly progressive cognitive and psychiatric syndromes, often affecting previously healthy individuals [1,2,3,4]. Importantly, many of these conditions are potentially reversible when diagnosed early [1,5], whereas delayed recognition is consistently associated with worse neurological outcomes [6,7] and, in paraneoplastic disease, missed opportunities for timely tumor detection.

From a diagnostic perspective, AE and PNS represent a paradigm of complex neuroimmunological disorders in which conventional clinical, imaging, and cerebrospinal fluid (CSF) findings are often insufficient, particularly in early disease stages. A substantial proportion of patients may present with normal magnetic resonance imaging (MRI) or only subtle CSF abnormalities, and a subset remains seronegative despite strong clinical suspicion. These challenges have shifted the diagnostic focus toward advanced laboratory-based approaches and biomarker-driven strategies.

This review is written deliberately from the perspective of laboratory medicine, a vantage point that remains underrepresented in the predominantly clinical and neuroimmunological literature on AE and PNS yet is decisive for diagnostic accuracy. Laboratory investigations are widely regarded as central to a substantial proportion of clinical decisions across medicine, although the frequently cited “70% claim” should be interpreted cautiously; their value derives not from test volume but from appropriate test selection, methodologically sound measurement, and context-aware interpretation [8]. In autoimmune neurology this principle is especially consequential: the diagnostic performance of neural antibody testing depends critically on the specimen tested (serum versus CSF), the combination of assay methods (tissue-based and cell-based screening with confirmatory steps), and the pretest probability defined by the clinical phenotype [9]. When testing is uncoupled from this laboratory-medicine framework—for example, single-method serum testing in low-probability patients—false-positive results are common and drive misdiagnosis, unnecessary immunosuppression, and missed alternative diagnoses; recent yield audits report low true-positive yield and frequent false-positive results, particularly when testing is performed in poorly selected populations or serum-only contexts [10]. A staged, phenotype-driven, dual-method testing strategy interpreted within its analytical and clinical context is therefore not a technical detail but the central determinant of whether these reversible disorders are recognized correctly and early. This laboratory-medicine lens is the unifying thread of the present review.

Over the past decade, the field has undergone a profound transformation driven by the expanding spectrum of neuronal and glial autoantibodies [11,12], improvements in assay methodologies including tissue-based assays (TBAs) and cell-based assays (CBAs) [13], and the introduction of quantitative biomarkers of neuroaxonal injury, most notably neurofilament light chain (NfL) [14,15]. The updated 2021 PNS-Care diagnostic criteria introduced an objective scoring system that, for combined definite and probable diagnoses, showed a sensitivity of 93% and a specificity of 100% in a retrospective single-center cohort of 484 patients with suspected PNS, compared with 67% and 99% for the 2004 criteria [16,17,18]; these are cohort-specific estimates that may not apply to unselected populations or to all AE/PNS presentations. In parallel, CSF cytokine and chemokine profiles are being investigated as adjunctive markers of intrathecal inflammation, particularly in antibody-negative cases, although their patterns overlap between autoimmune and infectious disorders and validated cutoffs are lacking [19,20,21].

Beyond individual biomarkers, there is growing emphasis on integrative diagnostic models that combine serological, CSF, and imaging data with emerging multi-omics technologies and artificial intelligence (AI)-based analytical tools. A new and critically important clinical challenge is the recognition and management of neurological adverse events triggered by immune checkpoint inhibitors (ICIs), which closely resemble classic PNS but require severity-adapted management within multidisciplinary oncological care [22,23,24].

The aim of this review is to provide a focused, biomarker-oriented overview of innovative diagnostic strategies for the early detection of autoimmune and paraneoplastic disorders of the nervous system. Particular emphasis is placed on the clinical integration of autoantibody testing, fluid biomarkers, and emerging multi-omics and computational approaches, with the goal of improving diagnostic accuracy and enabling earlier therapeutic intervention.

### Search Strategy and Selection Criteria

This narrative review was based on a structured search of PubMed/MEDLINE for English-language articles published between 1 January 2014 and 31 August 2026. Disease terms (“autoimmune encephalitis”, “paraneoplastic neurological/neurologic syndrome(s)”) were combined with methodological and biomarker terms (“neuronal antibody”, “neural antibody”, “cell-based assay”, “tissue-based assay”, “neurofilament light”, “cerebrospinal fluid”, “biomarker”, “PhIP-Seq”/“phage immunoprecipitation”, “immune checkpoint inhibitor”, “machine learning”, and “artificial intelligence”) in titles and abstracts; the full PubMed search string is given in Appendix A. Inclusion criteria were (i) original clinical or laboratory studies, diagnostic-accuracy and validation studies, systematic reviews and meta-analyses, consensus criteria, and guidelines; and (ii) relevance to laboratory diagnosis, assay methodology, fluid biomarkers, or diagnostic decision-making in AE or PNS. Exclusion criteria were single case reports (unless describing a newly identified antibody), conference abstracts, non-peer-reviewed material, and articles without laboratory or diagnostic content. The documented PubMed search (re-executed on 26 October 2026 for reproducibility) identified 1752 records. Titles and abstracts were screened, and approximately 250 articles were assessed in detail for eligibility, by four authors (D.S., D.Z., Z.Š. and P.K.). As in other narrative reviews, no formal risk-of-bias assessment was performed, and selection bias cannot be fully excluded. In total, 58 publications are cited, including foundational studies published before 2014 and clinical guidance identified through manual screening of reference lists, which were retained where they define established concepts, methods, or criteria. Recent laboratory-medicine-oriented syntheses and harmonization surveys were incorporated to reflect the current analytical landscape [25,26].

## 2. Immunopathological Basis of Autoimmune and Paraneoplastic Neurological Disorders

AE and PNS encompass both antibody-mediated and predominantly T-cell-mediated immune processes, with distinct implications for reversibility, cancer association, and therapeutic responsiveness.

### 2.1. Antibody-Mediated Versus T-Cell-Mediated Mechanisms

Antibodies targeting neuronal cell-surface or synaptic antigens—including N-methyl-D-aspartate receptor (NMDAR), leucine-rich glioma-inactivated protein 1 (LGI1), contactin-associated protein-like 2 (CASPR2), α-amino-3-hydroxy-5-methyl-4-isoxazolepropionic acid receptor (AMPAR), and γ-aminobutyric acid type B receptor (GABABR)—cause potentially reversible neuronal dysfunction through receptor internalization or altered synaptic signaling [1,12]. These disorders typically respond well to immunotherapy when diagnosed early [1,5,7].

In contrast, classic paraneoplastic antibodies directed against intracellular antigens (e.g., Hu/ANNA-1, Yo/PCA-1, Ri, Ma2, CV2/CRMP5, amphiphysin) primarily act as biomarkers of cytotoxic T-cell-mediated neuronal injury, often predicting irreversible neuronal loss and limited response to immunosuppression [11,27]. Anti-IgLON5 disease represents a unique paradigm bridging autoimmunity and neurodegeneration, with antibodies targeting the cell-surface IgLON5 protein leading to cytoskeletal disruption and secondary tauopathy [28,29,30].

### 2.2. Tumor–Immune Interactions and Diagnostic Implications

In PNS, ectopic expression of neuronal antigens by tumor cells breaks immune tolerance, triggering immune responses that cross-react with the nervous system [11]. Antigen localization informs both cancer risk and therapeutic expectations: high-risk intracellular antibodies mandate intensive and repeated tumor screening, whereas many cell-surface antibodies are associated with lower malignancy risk but excellent immunotherapy responsiveness when recognized early. Immunopathological classification is therefore central to diagnostic risk stratification and clinical decision-making.

## 3. Autoantibody Testing in Early Diagnosis

Autoantibody testing represents the cornerstone of laboratory diagnosis in AE and PNS. However, its clinical utility depends not only on analytical performance but also on appropriate test selection, timing, and interpretation within a phenotype-driven diagnostic framework.

### 3.1. Spectrum of Neuronal and Glial Autoantibodies

Autoantibodies can be broadly categorized into those targeting cell-surface/synaptic antigens and those directed against intracellular antigens, with distinct implications for pathogenesis, malignancy risk, and treatment responsiveness. Cell-surface antibodies—including NMDAR, LGI1, CASPR2, AMPAR, GABABR, DPPX, and IgLON5—are typically pathogenic and mediate potentially reversible neuronal dysfunction through receptor internalization or synaptic disruption. These disorders are strongly associated with favorable responses to early immunotherapy and exhibit relatively characteristic clinical phenotypes, such as faciobrachial dystonic seizures in LGI1 encephalitis [31] or prominent psychiatric manifestations in NMDAR encephalitis [1,7]. For LGI1/CASPR2-associated disease, the first randomized double-blind placebo-controlled trial demonstrated superiority of intravenous immunoglobulin over placebo in reducing seizure frequency, although the trial was terminated early because of slow enrollment and did not reach its planned sample size [32].

Intracellular onconeural antibodies (Hu, Yo, Ri, Ma2, CV2/CRMP5, amphiphysin, and SOX1) are most often associated with PNS and underlying malignancy and primarily reflect cytotoxic T-cell-mediated injury. Antibodies to glutamic acid decarboxylase 65 (GAD65), although intracellular, are associated with a comparatively low frequency of malignancy, in contrast to classic onconeural antibodies, and are best regarded as lower-risk markers requiring high titers and cautious interpretation [16]. From a diagnostic perspective, this distinction provides a biologically grounded framework for risk stratification and guides both oncologic screening and therapeutic urgency.

Antibodies to glial fibrillary acidic protein (GFAP) define autoimmune GFAP astrocytopathy, an increasingly recognized entity that typically presents as meningoencephalomyelitis, often with characteristic linear perivascular radial gadolinium enhancement on MRI and a variable association with neoplasia (e.g., ovarian teratoma). GFAP-IgG is most reliably detected in CSF by cell-based and tissue-based assays, and its inclusion in modern neural antibody panels improves recognition of this distinct astrocytopathy [33,34].

Recent years have witnessed rapid identification of novel autoantibodies through advanced discovery techniques. Phage immunoprecipitation sequencing (PhIP-Seq) incubates patient serum or CSF with a bacteriophage library displaying overlapping 36-amino-acid peptides that span the human proteome; antibody-bound phages are immunoprecipitated and identified by high-throughput sequencing [35]. Because the library displays short peptides, the method is best suited to linear epitopes and may miss conformational cell-surface targets; it is a research-based discovery tool whose candidate antibodies require orthogonal confirmation (e.g., TBA and CBA with the full-length antigen) and clinical validation before diagnostic use [35,36,37]. PhIP-Seq enabled the identification of anti-KLHL11, an intracellular antibody targeting Kelch-like protein 11, initially described in men with testicular seminomas presenting with a brainstem-cerebellar syndrome (rhombencephalitis), though the clinical spectrum has since broadened to include other germ cell tumors, female patients, and diverse malignancies; a recent cohort of 17 patients identified by a Dutch national reference laboratory further detailed the clinical manifestations, tumor associations, and long-term outcomes of anti-KLHL11 encephalitis [36,38,39]. Similarly, SKOR2-IgG was identified via PhIP-Seq in an initial report of two patients with lung and gallbladder adenocarcinoma, respectively, and requires confirmation in larger cohorts [37]. These discoveries can resolve the etiology in a subset of patients previously labeled as seronegative and underscore the evolving nature of the autoantibody repertoire (Table 1).

### 3.2. Diagnostic Algorithms and Testing Strategies

A key advance in recent years has been the shift from isolated antibody testing toward structured, phenotype-driven diagnostic algorithms. The 2021 PNS-Care criteria introduced a scoring system (PNS-Care Score) that integrates clinical phenotype, antibody type, presence or absence of cancer, and follow-up duration to classify patients into definite, probable, and possible PNS [16]. A retrospective validation study of 484 patients with suspected PNS evaluated at a single tertiary center reported, for the combined definite and probable categorization (PNS-Care Score ≥ 6), a sensitivity of 93% and a specificity of 100%, compared with 67% and 99% for the “definite” category of the 2004 criteria [16,17]. These are cohort-specific estimates from a selected referral population that excluded ICI-related presentations and syndromes not covered by the 2021 criteria, and they should not be generalized to unselected populations or to all AE/PNS presentations; notably, 15 patients with a PNS-Care Score ≤ 5 were judged likely to have PNS, most often KLHL11-IgG brainstem encephalitis [17]. These pooled figures should not be misread as the performance of the “definite” category alone, which is more specific but less sensitive.

For suspected AE, current expert consensus recommends a phenotype-driven, multi-step approach: (1) define the clinical phenotype; (2) perform first-line investigations (MRI, CSF analysis, electroencephalography) to establish an inflammatory context; (3) select targeted antibody panels based on phenotype and cancer risk profile; (4) apply a combined TBA–CBA testing strategy; and (5) integrate results with pretest probability [2,5,33,40]. TBAs using indirect immunofluorescence on rodent brain sections provide high sensitivity and enable recognition of characteristic staining patterns, including previously unrecognized antibodies, whereas CBAs offer high specificity by confirming antigen-specific binding, particularly for conformational cell-surface epitopes [25,40].

A complementary two-tiered TBA–CBA strategy is recommended to maximize diagnostic accuracy, with TBA screening followed by antigen-specific CBA confirmation [40]. A recent prospective comparison of commercial and in-house TBA and CBA platforms further demonstrated substantial inter-platform variability in performance for several antibodies, underscoring the need for assay standardization [13]. The diagnostic performance of antibody testing also differs by specimen compartment. For several antibodies—most notably NMDAR—CSF testing demonstrates superior sensitivity and specificity compared with serum; therefore, combined serum and CSF analysis is recommended whenever feasible [41,42].

### 3.3. Pitfalls and Sources of Misdiagnosis

Despite advances in assay technology, misinterpretation of autoantibody results remains a major clinical problem. It has recently been highlighted that increased awareness of AE has led to two unintended consequences [43]: a high frequency of misdiagnoses and the inappropriate use of diagnostic criteria for antibody-negative disease. Misdiagnoses typically arise from: (1) testing in low pretest probability settings, leading to incidental antibody detection without clinical relevance; (2) overinterpretation of low-titer antibodies, especially GAD65 or CASPR2 in serum; (3) use of incomplete assay panels (e.g., line blot-only strategies); (4) failure to confirm unexpected results with TBAs or repeat testing; (5) exclusive reliance on serum testing in conditions where CSF is more informative; and (6) ignoring alternative diagnoses such as neurodegenerative or psychiatric disorders.

Seronegative AE represents an additional diagnostic challenge. The criteria for probable antibody-negative AE require rigorous exclusion of alternative etiologies and well-substantiated absence of neural antibodies in both CSF and serum, ideally using both TBAs and CBAs covering a broad antigen range [2,33,43]. Accurate classification of these cases is essential for identifying homogeneous populations for treatment trials and outcome studies.

## 4. Fluid Biomarkers Beyond Autoantibodies

Fluid biomarkers have emerged as essential complementary tools in the diagnostic workup of AE and PNS, particularly in cases where autoantibody findings are negative, inconclusive, or difficult to interpret. In contrast to autoantibodies, which primarily provide etiological specificity, fluid biomarkers reflect key pathophysiological processes, including neuroinflammation and neuroaxonal injury, and therefore contribute to disease stratification and monitoring.

### 4.1. Neurofilament Light Chain

NfL is an analytically well-characterized biomarker of neuroaxonal damage across neurological disorders [14,15]. The introduction of ultrasensitive immunoassays, such as single-molecule array (Simoa) technology, has enabled reliable quantification of NfL in both CSF and serum, facilitating its implementation in clinical practice [14,44]. In multiple sclerosis and several neurodegenerative disorders, serum/plasma and CSF NfL concentrations correlate with disease activity, severity, and long-term outcome [14,15,44]; in AE, however, the evidence is more limited and less consistent, as summarized below.

A 2025 systematic review and meta-analysis evaluating NfL in neuronal surface antibody-associated AE (NSAE) demonstrated that both serum NfL (standardized mean difference [SMD] 0.909; 95% CI, 0.536–1.282) and CSF NfL (SMD 0.897; 95% CI, 0.508–1.286) were significantly elevated in patients with NSAE compared with controls [45]. However, the meta-analysis also noted that NfL levels were not consistently associated with disease severity, prognosis, or MRI/electroencephalographic abnormalities in NSAE, highlighting important differences from multiple sclerosis and suggesting that neuroaxonal injury, while present, may not be the predominant pathological feature. In anti-NMDA receptor encephalitis, serum NfL levels are elevated and correlate with neuroaxonal damage; however, their independent prognostic value at diagnosis remains limited, as any association with long-term outcome appears largely confounded by age [46]. In a 2026 multicenter prospective cohort of 92 patients with anti-NMDAR, anti-LGI1, or anti-CASPR2 encephalitis, CSF NfL z-scores were associated with baseline disease severity, and longitudinal NfL changes with severity and disability at follow-up [21]; these findings await independent replication. Taken together, NfL elevation in AE is well supported as a marker of neuroaxonal injury, whereas its prognostic and treatment-monitoring applications remain insufficiently validated.

From a laboratory standpoint, NfL interpretation requires age-adjusted reference ranges, strict standardization of sample handling, and awareness of inter-assay variability [14,15]. NfL lacks disease specificity, as levels rise across a broad range of neurological disorders, and should therefore be interpreted in the context of clinical phenotype, neuroimaging findings, and complementary laboratory biomarkers.

### 4.2. CSF Inflammatory Markers and Cytokine Profiles

Traditional CSF parameters—pleocytosis, elevated protein, and oligoclonal bands—remain important indicators of central nervous system (CNS) inflammation but lack sufficient sensitivity and specificity for early diagnosis. A subset of patients with AE may present with entirely normal CSF findings in the early disease phase.

Cytokine and chemokine profiling has been explored as a means of characterizing intrathecal inflammation. A systematic review of 83 studies found overlapping cytokine/chemokine patterns across neuroinflammatory syndromes, with some trends—B-cell-related markers such as CXCL13 and B-cell activating factor (BAFF) predominantly elevated in autoantibody-associated disorders, and interferon-γ mainly in viral encephalitis—but no disease-specific signature or validated cutoff [19]. A 2026 cross-sectional study of 43 patients with AE (29 seronegative) reported higher CSF CXCL10, CXCL13, IL-6, IL-13, and other cytokines than in 34 controls, with comparable profiles in seronegative and antibody-positive disease [20]. However, the control group comprised 33 patients with idiopathic intracranial hypertension and only one patient with viral encephalitis; the study therefore cannot establish discrimination of AE from infectious encephalitis, and its authors presented the findings as exploratory [20]. In a 2026 prospective multicenter cohort that included varicella-zoster virus encephalitis controls, cytokines showed limited diagnostic or prognostic utility [21]. CSF cytokine and chemokine profiles should therefore be regarded as investigational markers of intrathecal immune activation that lack standardized platforms, validated cutoffs, and adequate infectious-disease comparator data, rather than as diagnostic discriminators.

### 4.3. Intrathecal Immunoglobulin Synthesis: Kappa Free Light Chains

Intrathecal kappa free light chain (KFLC) synthesis, assessed in paired CSF–serum samples, has emerged as a sensitive adjunctive marker of intrathecal humoral activity. In the 2026 prospective cohort described above, intrathecal KFLC synthesis was detected in 94% of patients with anti-NMDAR, 50% with anti-LGI1, and 53% with anti-CASPR2 encephalitis, exceeding the sensitivity of CSF-restricted oligoclonal bands or pleocytosis; specificity was moderate (44%) against pooled control groups that included multiple sclerosis and viral encephalitis, but reached 90% against non-inflammatory neurological controls [21]. KFLC therefore supports, but does not establish, an inflammatory context and cannot by itself discriminate AE from other inflammatory or infectious CNS disorders.

### 4.4. Complement, Soluble GFAP, and Other Emerging Biomarkers

Complement activation has been investigated as a potential marker of humoral immune activity in antibody-mediated neurological disease; however, its pathogenic role appears disease-specific. In anti-NMDAR encephalitis, brain analyses did not support a major complement-mediated mechanism, arguing against broad clinical utility of complement markers in this specific disorder [47]. Soluble GFAP, a marker of astrocytic injury measurable by Simoa, has also been studied in AE. In a cohort of 42 patients (31 with active and 11 with stable disease), CSF GFAP correlated with the albumin quotient (r = 0.59) and IgG quotient (r = 0.55) and weakly with CSF cell count (r = 0.29); serum NfL (r = 0.41) and serum GFAP (r = 0.30) correlated with the Clinical Assessment Scale in Autoimmune Encephalitis, and the serum NfL/GFAP ratio was higher in active than in stable AE, whereas CSF GFAP and NfL did not differ according to epileptic activity or inflammatory MRI lesions [48]. GFAP showed limited diagnostic or prognostic utility in the larger 2026 prospective cohort [21], so soluble GFAP should be regarded as an emerging, not yet validated marker. It must be distinguished from GFAP-IgG, which remains the defining biomarker of autoimmune GFAP astrocytopathy [33,34]. Emerging markers such as YKL-40 (chitinase-3-like protein 1) reflect microglial and astrocytic activation and have been associated with disease remission in AE, but require further prospective validation [49].

### 4.5. Integrated Use of Fluid Biomarkers

The greatest diagnostic value of fluid biomarkers lies in their integration with autoantibody testing, neuroimaging, and clinical phenotype. Combined biomarker approaches may: (1) support an inflammatory or neuroaxonal-injury context in antibody-negative cases with high clinical suspicion; and (2) provide adjunctive information on disease activity. The use of NfL, GFAP, or other fluid biomarkers to guide treatment intensity, monitor treatment response, or detect early relapse in AE has not yet been validated and should currently be confined to research settings. Future diagnostic strategies will increasingly rely on multimarker panels rather than single biomarkers, necessitating assay standardization, age-adjusted reference ranges, and prospective validation in larger cohorts. Table 2 summarizes, for each biomarker, the sample type, intended use, and analytical availability separately from clinical validation status; apart from conventional CSF parameters, GFAP-IgG, and NfL as a marker of neuroaxonal injury, the listed markers should be regarded as investigational in AE.

## 5. Multi-Omics and Artificial Intelligence Approaches

Recent advances in high-throughput technologies have enabled the application of multi-omics approaches—including proteomics, transcriptomics, and immune repertoire sequencing—to the study of AE and PNS. These methods provide an unbiased framework for identifying molecular signatures associated with neuroinflammation, synaptic dysfunction, and tumor-driven immune responses.

PhIP-Seq, described in Section 3.1, is the most advanced example of such discovery-oriented profiling in AE/PNS [36,37]. Like other multi-omics methods, it remains a research tool: findings are typically derived from small, highly selected referral cohorts, require orthogonal confirmation, and have not been evaluated prospectively for diagnostic yield in unselected patients.

From a laboratory perspective, proteomic, transcriptomic, and immune-repertoire analyses are hypothesis-generating tools for studying antigen-driven B- and T-cell responses [11] and may eventually help characterize seronegative cases. Their clinical relevance to AE/PNS diagnostics, however, is not established: analytical validity (reproducibility, batch effects, and sensitivity to pre-analytical conditions) is rarely reported, cohorts are small and selected, the number of measured features greatly exceeds the number of patients—creating a high risk of overfitting—and no multi-omics signature has been validated prospectively.

AI-based analytical models have recently been proposed to integrate heterogeneous data sources, including clinical features, neuroimaging, CSF parameters, and serological findings. A single retrospective two-center study developed an AI multiclass model that classified the etiology of meningitis and encephalitis (autoimmune, bacterial, viral, and tuberculous) from clinical and laboratory variables available within 24 h of admission; in an external validation cohort of 220 patients, it achieved an area under the receiver operating characteristic curve (AUROC) of 0.916 and outperformed three clinicians in that study [50]. This estimate derives from one retrospective dataset, in which the etiological reference standard was itself based on CSF laboratory results, and has not been confirmed prospectively or in other populations. Separately, MRI-based machine learning approaches have been explored for predicting antibody serostatus in suspected AE [51]. However, most studies remain retrospective, involve small and highly selected cohorts, are at risk of overfitting, and none has yet demonstrated an effect on diagnostic or patient outcomes. Prospective, multicenter validation and attention to model interpretability are essential before AI tools can be safely integrated into clinical workflows.

## 6. Neuroimaging Patterns Supporting Early Diagnosis

MRI abnormalities, including fluid-attenuated inversion recovery (FLAIR) hyperintensities, may occur in both AE and infectious encephalitis. These findings show considerable overlap and cannot, in isolation, reliably distinguish between the two conditions [2,52]. Electroencephalographic findings often support an autoimmune etiology when combined with appropriate clinical features [52,53]. Specific patterns—such as mesial temporal involvement, brainstem–diencephalic lesions, or longitudinally extensive spinal cord lesions—may suggest particular antibody profiles and guide targeted testing. Radiologists can be among the first to suspect AE and trigger a comprehensive neuroimmunological workup.

18F-fluorodeoxyglucose positron emission tomography (18F-FDG PET) demonstrates high sensitivity (pooled sensitivity ~87% in a systematic review and meta-analysis), frequently revealing characteristic metabolic patterns when MRI is normal or inconclusive [54], and exhibits antibody-specific hypometabolic or hypermetabolic signatures [55,56]. PET can help differentiate AE from neurodegenerative dementias and support early immunotherapy initiation in patients with rapidly progressive cognitive decline and atypical MRI findings.

## 7. Immune Checkpoint Inhibitor-Related Neurological Syndromes

Immune checkpoint inhibitors (ICIs)—including anti-PD-1, anti-PD-L1, and anti-CTLA-4 agents—have revolutionized cancer treatment but can trigger a broad spectrum of neurological adverse events (NAEs) that closely resemble classic PNS. The incidence of neurological immune-related adverse events (irAEs) has been reported between approximately 1% and 12%, depending on the agent, combination regimen, ascertainment method, and definition used; severe (grade ≥ 3) events are uncommon and generally occur in fewer than 1% of treated patients [22,23,57].

The clinical spectrum of ICI-related neurotoxicity encompasses myasthenia gravis, myositis, encephalitis, peripheral neuropathies, meningitis, and myelitis. A particularly important overlap syndrome is the triad of myocarditis, myasthenia gravis, and myositis (the “3M syndrome”), which carries high mortality and requires urgent recognition [22]. Encephalitis occurring after ICI therapy may present with or without detectable neural antibodies. Some patients harbor classical paraneoplastic antibodies (e.g., Hu, Ma2), which may have been unmasked by immune checkpoint blockade, while others have novel or unidentified antibody specificities.

From a laboratory diagnostic perspective, ICI-related NAEs pose unique challenges. The 2021 PNS-Care criteria include specific recommendations for ICI-triggered syndromes, acknowledging that a substantial proportion of cases remain seronegative despite comprehensive antibody screening [16,22]. NfL has emerged as a promising, but still insufficiently validated, biomarker in this context. Elevated NfL may reflect neuroaxonal injury in cancer patients with acute neurological complications, including PNS and ICI-related neurotoxicity, while longitudinal NfL monitoring could potentially assist in assessing recovery and treatment response [45,57].

Management of the neurological immune-related adverse event should be distinguished from management of the underlying malignancy. For the adverse event itself, management is graded by severity: ICI therapy is usually withheld temporarily for moderate events, with possible resumption after resolution, whereas permanent discontinuation is generally advised for severe (grade 3–4) events and for specific syndromes such as myasthenia gravis, Guillain–Barré syndrome, or transverse myelitis; corticosteroids are first-line treatment, with intravenous immunoglobulin or plasma exchange in severe or refractory cases [22,24]. Decisions on rechallenge and on further cancer treatment are made within multidisciplinary neuro-oncological care, in which oncological treatment remains an integral component [22,23,24]. The distinction between ICI-triggered PNS (where tumor immunotherapy unmasks pre-existing antitumor immunity) and direct ICI neurotoxicity has important prognostic and therapeutic implications. Early clinical recognition supported by appropriate laboratory testing is critical, whereas the value of systematic biomarker-based surveillance in patients receiving ICIs has not yet been established.

## 8. Challenges in Laboratory Standardization and Clinical Implementation

Substantial inter-laboratory variability exists in assay platforms, antigen constructs, cutoffs, and reporting standards for neural autoantibody testing. A recent European survey of laboratories performing PNS autoantibody detection confirmed considerable heterogeneity in assay execution, reporting, and quality control, and highlighted the need for laboratory-specific recommendations to harmonize practices and improve diagnostic quality [26]. External quality assurance programs and harmonization initiatives are essential to ensure reproducibility, particularly for cell-based assays where fixation, cell substrates, and cut-off definitions vary widely. Recent laboratory-medicine syntheses have reinforced the importance of appropriate patient selection, pre-analytical control, and integrated serum plus CSF testing pathways in reducing false-positive and false-negative results [25].

In current practice, paired serum and CSF should be submitted for neural antibody testing in suspected AE, because some antibodies (e.g., LGI1 and CASPR2) are more sensitively detected in serum with commercially available assays, whereas others (e.g., NMDAR and GFAP) are more sensitively and specifically detected in CSF; serum-only NMDAR-IgG positivity with negative CSF is a recognized warning sign of a false-positive result [25,40]. Samples should ideally be collected in the acute phase and before immunotherapy, because plasma exchange or neonatal Fc receptor inhibitors may cause false-negative results and intravenous immunoglobulin may cause low-level false-positive results (e.g., GAD65), which can be clarified by repeat testing after 3–6 months [25]. Specimens should be refrigerated at approximately 4 °C for short-term storage or frozen at ≤−20 °C when testing is delayed, avoiding repeated freeze–thaw cycles [25]. Assay-specific pre-treatment should be documented by the laboratory; for example, some in-house live-cell CBAs heat-inactivate serum before testing (35 min at 56 °C in a clinically validated flow-cytometric AQP4-IgG assay), a step that inactivates complement [58].

Cutoffs and titers are method-specific. CBAs and TBAs are usually reported qualitatively at a laboratory-defined screening dilution, which differs among laboratories—in a recent European Autoimmunity Standardisation Initiative (EASI) survey of 139 laboratories, only 61% were aligned on sample dilution [26]—and endpoint titration helps to interpret weak or isolated serum positivity (e.g., CASPR2 or MOG) [25]. For GAD65, only high concentrations support neurological autoimmunity, and thresholds differ by method (e.g., >20 nmol/L by radioimmunoassay versus >10,000 IU/mL in serum and >100 IU/mL in CSF by enzyme-linked immunosorbent assay), whereas low concentrations occur in type 1 diabetes and in healthy individuals [25]. Isolated line-blot positivity for intracellular antibodies without corresponding TBA staining has a positive predictive value below 50% and should not be reported as diagnostic without confirmation [25]. Antibody titers correlate imperfectly with disease activity, so repeat testing for disease monitoring is not established, although CSF NMDAR titers relate more closely to outcome than serum titers [25,42].

Testing should be performed in accredited laboratories participating in external quality assessment (EQA); in the EASI survey, 46% (indirect immunofluorescence) and 42% (line/dot blot) of laboratories held ISO 15189 [59] accreditation, and 43% participated in more than one EQA program [26]. In our view, a standardized report should state the specimen type, the methods used (TBA; fixed or live CBA; line blot; enzyme-linked immunosorbent assay or radioimmunoassay), the screening dilution or titer, the result of confirmatory testing, and an interpretive comment relating the result to the pretest probability, with recommendations for CSF or reference-laboratory confirmation where appropriate. Direct communication between laboratory specialists and clinicians—through interpretive comments, discussion of discordant results, and agreed reflex and send-out algorithms—remains the principal mechanism for correlating laboratory results with the clinical evaluation [25,40].

For emerging fluid biomarkers, harmonization is similarly critical. NfL quantification requires standardized pre-analytical protocols, certified reference materials, and age-adjusted reference intervals to enable meaningful cross-study and cross-center comparisons [14,15]. Cytokine profiling faces analogous challenges, with no consensus on optimal platforms, sample handling requirements, or clinically validated cutoff values.

Reference centers play an indispensable role in confirmatory testing, interpretation of rare antibodies, and access to experimental biomarkers and emerging techniques such as live neuron studies. Clinically, over-testing in low pretest probability settings risks incidental positives and inappropriate immunosuppression, whereas under-testing perpetuates diagnostic delay. Evidence-based diagnostic pathways that integrate pretest probability, staged testing, and clear triggers for immunotherapy are essential, alongside health-economic evaluations of advanced biomarker and imaging strategies.

## 9. Future Directions and Translational Perspectives

Integrated panels combining autoantibodies, NfL, intrathecal KFLC synthesis, and other CSF markers with MRI/18F-FDG PET features may outperform single tests for early AE/PNS diagnosis and cancer-risk stratification, but this hypothesis requires prospective testing. Personalized diagnostic algorithms tailored to age, syndrome, and comorbidities can optimize test selection and timing and could be implemented as clinical decision-support tools in electronic health records.

Continued application of PhIP-Seq and other unbiased serological discovery methods will likely identify additional autoantibody targets, which, once confirmed by orthogonal assays and clinically validated, may further reduce the proportion of seronegative cases and refine risk stratification [36,37]. Point-of-care and rapid assay platforms for key biomarkers such as NfL may enable faster triage in acute settings.

The expanding use of ICIs in oncology demands dedicated biomarker surveillance programs and prospective registries to characterize the spectrum and predictors of neurological irAEs [22,23]. Development of predictive biomarkers for ICI-related neurotoxicity represents an unmet clinical need.

Prospective multicenter studies should validate biomarker thresholds, AI-based prediction models, and imaging-guided diagnostic pathways, with outcomes including time to diagnosis, treatment initiation, and long-term disability. Standardization of biomarker assays and harmonization of diagnostic algorithms across centers will be critical for translating these advances into routine clinical practice. These efforts will underpin the transition toward precision neuroimmunology.

## 10. Conclusions

Early, accurate diagnosis of autoimmune and paraneoplastic neurological disorders is critical to prevent irreversible neurological disability and enable timely tumor detection. The 2021 PNS-Care criteria provide an objective framework for diagnostic certainty; their high sensitivity and specificity for combined definite and probable diagnoses were demonstrated in a retrospective, selected single-center cohort and require confirmation in unselected populations [16,17]. Phenotype-driven autoantibody testing using combined TBA–CBA strategies, complemented by NfL as a marker of neuroaxonal injury, conventional CSF inflammatory parameters, intrathecal KFLC synthesis, and MRI/18F-FDG PET, provides the current diagnostic platform for earlier recognition, whereas cytokine profiles, soluble GFAP, and prognostic use of NfL remain investigational.

Novel autoantibody discovery through PhIP-Seq has expanded the recognized antigen repertoire, resolving a subset of previously seronegative cases [36,37,38], while early AI-assisted models, so far evaluated only retrospectively, may in future help integrate complex multimodal data [50,51]. The growing recognition of ICI-related neurological syndromes as a distinct clinical entity demands heightened clinical vigilance and appropriate laboratory testing in oncology settings.

Crucially, the impact of every one of these advances is realized only through the discipline of laboratory medicine: the diagnostic value of neural antibodies, NfL, CSF inflammatory markers, and emerging multi-omics or AI-derived signatures depends on appropriate test selection guided by pretest probability, dual-method analytical confirmation, and interpretation within the clinical context rather than on test availability alone [8,9]. Failure to apply this framework—particularly reliance on single-method serum testing in low-probability patients—generates false-positive results that drive misdiagnosis and inappropriate immunosuppression, underscoring that a staged, phenotype-driven laboratory strategy is the principal safeguard of diagnostic accuracy [10]. Positioning AE and PNS diagnostics squarely within laboratory medicine—rather than treating biomarkers as isolated clinical add-ons—is, in our view, the most actionable lever for earlier and more reliable recognition.

Ultimately, the integration of multimodal biomarkers into clinically applicable, harmonized algorithms—supported by reference center networks and prospective validation studies—will be essential to achieve precision diagnostics in neuroimmunology and translate these advances into equitable, routine clinical care.

## Figures and Tables

**Table 1:** Major neuronal and glial autoantibodies, core syndromes, and malignancy risk (risk categories per PNS-Care 2021 criteria).

Autoantibody	Target Location	Typical Clinical Syndromes	Cancer Association	Risk Category (PNS-Care 2021)	Diagnostic Relevance
Anti-Hu (ANNA-1)	Intracellular	Sensory neuronopathy, encephalomyelitis, limbic encephalitis	High (SCLC)	High-risk	Strong paraneoplastic marker; mandates tumor search
Anti-Yo (PCA-1)	Intracellular	Paraneoplastic cerebellar degeneration	High (breast, gynecologic)	High-risk	Indicates urgent and repeated tumor screening
Anti-Ri (ANNA-2)	Intracellular	Brainstem encephalitis, opsoclonus–myoclonus	High (breast, SCLC)	High-risk	Paraneoplastic indicator
Anti-Ma2	Intracellular	Limbic/diencephalic encephalitis	High (testicular germ cell)	High-risk	Young males; testicular ultrasound
Anti-CV2/CRMP5	Intracellular	Chorea, neuropathy, encephalomyelitis	High (SCLC, thymoma)	High-risk	Multifocal PNS presentations
Anti-amphiphysin	Intracellular	Stiff-person syndrome, encephalomyelitis, sensory neuronopathy	High (breast, SCLC)	High-risk	Paraneoplastic marker; often coexists with other antibodies
Anti-SOX1	Intracellular	Lambert–Eaton myasthenic syndrome, cerebellar degeneration	High (SCLC)	High-risk	Paraneoplastic marker
Anti-KLHL11	Intracellular	Rhombencephalitis, cerebellar degeneration	High (seminoma; expanding spectrum)	High-risk	Discovered via PhIP-Seq; expanding clinical spectrum
Anti-SKOR2	Intracellular	Progressive cerebellar/brainstem syndrome	Reported adenocarcinoma associations in small series	Not yet established (provisional)	Novel PhIP-Seq-identified biomarker; risk category pending further cohorts
Anti-Tr (DNER)	Cell-surface	Paraneoplastic cerebellar degeneration	High (Hodgkin lymphoma)	High-risk	Strong association with Hodgkin lymphoma
NMDA receptor	Cell-surface	Encephalitis with psychiatric features, dyskinesias, seizures	Intermediate (ovarian teratoma)	Intermediate-risk	CSF testing preferred; early recognition critical
LGI1	Cell-surface	Limbic encephalitis, faciobrachial dystonic seizures	Low (~10%)	Low-risk	Excellent immunotherapy response
CASPR2	Cell-surface	Limbic encephalitis, Morvan syndrome, neuromyotonia	Low overall; intermediate in Morvan syndrome (thymoma)	Low-risk (Intermediate in Morvan syndrome)	Guides immunotherapy and cancer screening
AMPAR	Cell-surface	Limbic encephalitis	Intermediate (lung, breast, thymoma)	Intermediate-risk	Relapsing course; tumor search indicated
GABA-B receptor	Cell-surface	Limbic encephalitis with prominent seizures	Intermediate (mainly SCLC)	Intermediate-risk	Frequent SCLC association; tumor search mandatory
GABA-A receptor	Cell-surface	Encephalitis with refractory seizures/status epilepticus, multifocal MRI lesions	Low (occasional thymoma)	Low-risk	Severe but immunotherapy-responsive
mGluR1	Cell-surface	Cerebellar ataxia	Low (occasional Hodgkin lymphoma)	Low-risk	Rare; ataxia phenotype
mGluR5	Cell-surface	Limbic encephalitis (Ophelia syndrome)	Intermediate (Hodgkin lymphoma)	Intermediate-risk	Association with Hodgkin lymphoma
P/Q-type VGCC	Cell-surface	Lambert-Eaton myasthenic syndrome, cerebellar degeneration	Intermediate (SCLC, particularly in LEMS)	Intermediate-risk	Tumor search recommended, particularly in LEMS
DPPX	Cell-surface	Encephalitis with hyperexcitability, diarrhea, weight loss	Low	Low-risk	Chronic course; may respond to immunotherapy
IgLON5	Cell-surface	Sleep disorder, bulbar dysfunction, movement disorders, cognitive decline	Low	Low-risk	Unique autoimmune–neurodegenerative overlap; tau pathology
GAD65 (high titer)	Intracellular	Stiff-person spectrum, epilepsy, cerebellar ataxia	Low (<15%)	Low-risk	Limited pathogenic specificity; requires high titers
AQP4 (NMO-IgG)	Cell-surface	Neuromyelitis optica spectrum disorder	Low	Low-risk	Overlap/differential demyelinating disorder; defining biomarker of NMOSD; CBA recommended
MOG	Cell-surface	MOG antibody-associated disease (ADEM, optic neuritis, encephalitis)	Low	Low-risk	Overlap/differential demyelinating disorder; live CBA preferred
GFAP	Intracellular (astrocytic)	Autoimmune GFAP astrocytopathy (meningoencephalomyelitis)	Low overall (occasional ovarian teratoma, adenocarcinoma)	Low-risk	CSF testing superior to serum; characteristic radial perivascular enhancement

Abbreviations: ADEM, acute disseminated encephalomyelitis; AMPAR, α-amino-3-hydroxy-5-methyl-4-isoxazolepropionic acid receptor; ANNA, antineuronal nuclear antibody; AQP4, aquaporin-4; CASPR2, contactin-associated protein-like 2; CBA, cell-based assay; CRMP5, collapsin response-mediator protein 5; DNER, delta/notch-like epidermal growth factor-related receptor; DPPX, dipeptidyl-peptidase-like protein 6 (DPP6; also termed dipeptidyl aminopeptidase-like protein 6); GABA-A R, γ-aminobutyric acid type A receptor; GABA-B R, γ-aminobutyric acid type B receptor; GAD65, glutamic acid decarboxylase 65-kDa isoform; GFAP, glial fibrillary acidic protein; IgLON5, immunoglobulin-like domain-containing protein 5; KLHL11, Kelch-like protein 11; LEMS, Lambert-Eaton myasthenic syndrome; LGI1, leucine-rich glioma-inactivated protein 1; mGluR, metabotropic glutamate receptor; MOG, myelin oligodendrocyte glycoprotein; NMDAR, N-methyl-D-aspartate receptor; NMOSD, neuromyelitis optica spectrum disorder; PCA-1, Purkinje cell antibody type 1; PhIP-Seq, phage immunoprecipitation sequencing; PNS-Care, updated 2021 diagnostic criteria for paraneoplastic neurological syndromes [16]; SCLC, small-cell lung cancer; SKOR2, SKI family transcriptional corepressor 2; VGCC, voltage-gated calcium channel. Risk categories follow PNS-Care 2021: high-risk > 70%, intermediate-risk 30–70%, low-risk < 30% association with malignancy [16].

**Table 2:** Established and emerging fluid biomarkers in autoimmune and paraneoplastic neurological disorders.

Biomarker	Sample Type	Clinical Utility	Added Diagnostic Value	Key References	Analytical Availability/Clinical Validation Status
Neurofilament light chain (NfL)	CSF/serum	Marker of neuroaxonal injury; prognostic value in AE inconsistent	Sensitive marker of neuroaxonal injury; elevated in NSAE (SMD ~0.9 vs. controls)	[14,15,21,45,46]	Analytical: available on ultrasensitive platforms (e.g., Simoa); requires age-adjusted reference ranges. Clinical: not disease-specific; prognostic and monitoring use in AE not validated
CSF pleocytosis/oligoclonal bands	CSF	Inflammatory confirmation	Supports autoimmune etiology; part of diagnostic criteria	[2,5]	Analytical: widely available. Clinical: part of diagnostic criteria; limited sensitivity in early disease
Kappa free light chains (KFLC)	CSF (with paired serum)	Intrathecal humoral activity; supports an inflammatory context	High sensitivity (94% in anti-NMDAR encephalitis); moderate specificity against inflammatory and infectious controls	[21]	Analytical: available; standardized quotient-based calculations. Clinical: one prospective AE cohort; interpret with blood–CSF barrier integrity
CSF lactate	CSF	Differentiation of inflammatory vs. infectious/metabolic processes	Typically normal in autoimmune encephalitis; elevation favors bacterial/mitochondrial mimics	[5]	Analytical: widely available. Clinical: non-specific; useful as exclusionary marker
Cytokine/chemokine profiles (IL-21, IP-10/CXCL10, CXCL13, IL-6)	CSF	Investigational marker of intrathecal immune activation; possible adjunct in seronegative AE	Elevated versus non-inflammatory controls; comparable in seronegative and antibody-positive AE; patterns overlap with infectious encephalitis; discrimination from infectious mimics not established	[19,20,21]	Analytical: no standardized platform or cutoffs. Clinical: investigational; no validated infectious-disease comparator data
Complement activation markers (C3, C4, C5a)	CSF/serum	Immune activation assessment	Disease-specific; not supported as a general marker in NMDAR-AE (limited complement-mediated pathology)	[47]	Analytical: not standardized for this indication. Clinical: investigational; utility likely restricted to selected antibody-mediated disorders
Soluble GFAP	CSF/serum	Astrocytic injury; association with disease activity in AE	Correlates with CSF barrier and inflammatory indices; serum NfL/GFAP ratio higher in active than stable AE in one cohort; limited utility in a larger prospective cohort	[21,48]	Analytical: ultrasensitive platforms (e.g., Simoa). Clinical: emerging; not validated
GFAP-IgG	CSF/serum	Diagnostic biomarker of autoimmune GFAP astrocytopathy	Defining biomarker; CSF testing superior to serum	[33,34]	Analytical: CBA/TBA available; CBA-based confirmation preferred. Clinical: established diagnostic biomarker
YKL-40 (chitinase-3-like protein 1)	CSF	Glial activation/neuroinflammation	CSF YKL-40 evolution associated with AE remission; reflects microglial/astrocytic activation	[49]	Analytical: not standardized. Clinical: investigational; limited prospective data
Immune repertoire sequencing	Blood/CSF	Detection of clonal B/T-cell expansion	Identifies antigen-driven immune responses; aids biomarker discovery	[11,36]	Analytical: research use only. Clinical: not validated; future diagnostic integration

Abbreviations: AE, autoimmune encephalitis; CBA, cell-based assay; CSF, cerebrospinal fluid; GFAP, glial fibrillary acidic protein; IL-6, interleukin-6; IL-21, interleukin-21; IP-10, interferon-γ-induced protein 10; KFLC, kappa free light chains; NfL, neurofilament light chain; NMDAR-AE, N-methyl-D-aspartate receptor autoimmune encephalitis; NSAE, neuronal surface antibody-associated autoimmune encephalitis; Simoa, single-molecule array; SMD, standardized mean difference.

## Data Availability

No new data were created or analyzed in this study. Data sharing is not applicable to this article.

## Abbreviations

AE = autoimmune encephalitis; AI = artificial intelligence; AMPAR = α-amino-3-hydroxy-5-methyl-4-isoxazolepropionic acid receptor; ANNA-1 = antineuronal nuclear antibody type 1; CASPR2 = contactin-associated protein-like 2; CBA = cell-based assay; CRMP5 = collapsin response mediator protein 5; CSF = cerebrospinal fluid; CV2 = anti-CV2 antibody; DPPX = dipeptidyl-peptidase-like protein 6 (DPP6; also termed dipeptidyl aminopeptidase-like protein 6); EASI = European Autoimmunity Standardisation Initiative; EQA = external quality assessment; FDG-PET = fluorodeoxyglucose positron emission tomography; GABABR = γ-aminobutyric acid type B receptor; GAD65 = glutamic acid decarboxylase 65; GFAP = glial fibrillary acidic protein; ICI = immune checkpoint inhibitor; IgG = immunoglobulin G; IgLON5 = immunoglobulin-like cell adhesion molecule 5; KFLC = kappa free light chain; KLHL11 = Kelch-like protein 11; LGI1 = leucine-rich glioma-inactivated 1; MRI = magnetic resonance imaging; NfL = neurofilament light chain; NMDAR = N-methyl-D-aspartate receptor; PCA-1 = Purkinje cell cytoplasmic antibody type 1; PhIP-Seq = phage immunoprecipitation sequencing; PNS = paraneoplastic neurological syndrome; SKOR2 = SKI family transcriptional corepressor 2; SOX1 = SRY-box transcription factor 1; TBA = tissue-based assay.

## Appendix A

PubMed search string (publication dates 1 January 2014 to 31 August 2026; re-executed on 26 October 2026, 1752 records): (“autoimmune encephalitis”[tiab] OR “paraneoplastic neurological syndrome”[tiab] OR “paraneoplastic neurologic syndrome”[tiab] OR “paraneoplastic neurological syndromes”[tiab] OR “paraneoplastic neurologic syndromes”[tiab]) AND (“neuronal antibod*”[tiab] OR “neural antibod*”[tiab] OR “cell-based assay*”[tiab] OR “tissue-based assay*”[tiab] OR “neurofilament light”[tiab] OR “cerebrospinal fluid”[tiab] OR biomarker*[tiab] OR “PhIP-Seq”[tiab] OR “phage immunoprecipitation”[tiab] OR “immune checkpoint inhibitor*”[tiab] OR “machine learning”[tiab] OR “artificial intelligence”[tiab]) AND english[la].
